# Identification of suitable reference genes during the formation of chlamydospores in *Clonostachys rosea* 67‐1

**DOI:** 10.1002/mbo3.505

**Published:** 2017-07-05

**Authors:** Jun Zhang, Zhanbin Sun, Shidong Li, Manhong Sun

**Affiliations:** ^1^ Institute of Plant Protection Chinese Academy of Agricultural Sciences Beijing China

**Keywords:** actin, chlamydospore, *Clonostachys rosea*, reference gene, reverse transcription quantitative PCR, succinate‐semialdehyde dehydrogenase

## Abstract

*Clonostachys rosea* is a potential biocontrol fungus that can produce highly resistant chlamydospores under specific conditions. To investigate the genes related to chlamydospore formation, we identified reliable reference genes for quantification of gene expression in *C. rosea* 67‐1 during sporulation. In this study, nine reference genes, actin (*ACT*), elongation factor 1 (*EF1*), glyceraldehyde‐3‐phosphate dehydrogenase (*GAPDH*), histone (*HIS*), RNA polymerase II CTD phosphatase Fcp1 (*RPP*), succinate‐semialdehyde dehydrogenase (*SSD*), TATA‐binding protein (*TBP*), ubiquitin (*UBQ*), and ubiquitin‐conjugating enzyme (*UCE*), were selected and cloned from 67‐1, and their expression stability during chlamydospore formation was determined using reverse transcription quantitative PCR and assessed using the software geNorm, NormFinder and BestKeeper. The Ct values of the candidates ranged from 19.9 to 29.7, among which *HIS*,*ACT* and *SSD* exhibited high expression levels. The statistical analysis showed that *ACT* and *SSD* were most stably expressed, while *UBQ* and *GAPDH* showed relatively large variations under different culture conditions. Calculation of pairwise variation value indicated that two reference genes were required for precise quantification. Finally, *ACT* and *SSD* were selected to normalize gene expression during chlamydospore production in *C. rosea* 67‐1. To the best of our knowledge, this is the first report of *SSD* as a reference gene. This study will facilitate the accurate quantification of differentially expressed genes during the generation of chlamydospores and contribute to the investigation of the molecular mechanism underlying chlamydospore formation in *C. rosea*.

## 1 INTRODUCTION

*Clonostachys rosea* (syn. *Gliocladium roseum*) is a widely distributed mycoparasite, and it has shown great potential against various plant fungal pathogens through multiple mechanisms, such as mycoparasitism, competition, antagonism, and induction of plant resistance (Morandi, Maffia, Mizubuti, Alfenas, & Barbosa, 2003; Mouekouba et al., 2014; Ortiz & Orduz, 2001; Papavizas, 1985; Rodríguez, Cabrera, Gozzo, Eberlin, & Godeas, 2011). The conidia of *C. rosea*, which are easily obtained by mass production, are commonly used as the main components of biocontrol agents. However, the relatively low adversity resistance of the conidiospores may result in instability and a short shelf life of fungal biopesticides, limiting their large‐scale production and application. The chlamydospore is a type of vital resistant spore, and it is able to survive under unfavorable conditions (Armengol, Sales, & Garcia‐Jimenez, 1999; Beagle‐Ristaino & Papavizas, 1985; Caldwell, 1958; Eyal, Baker, Reeder, Devane, & Lumsden, 1997). Compared with conidia, chlamydospores are much larger and have a thicker cell wall, higher resistance to harsh environments, and equal biocontrol ability (Dong, Sun, Li, Peng, & Luo, 2014). Chlamydospores are of great significance in fungal survival as well as in the commercialization of fungal biopesticides. Several studies have revealed the nutrient and environmental conditions that influence chlamydospore formation in *C. rosea* (Li, Qu, Tian, & Zhang, 2005; Sun, Chen, Liu, Li, & Ma, 2014; Zhang, Sun, Zhang, Xie, & Li, 2010); however, the mechanism underlying chlamydospore formation has not yet been elucidated. Information on chlamydospore production‐related genes will facilitate the development and wide applications of *C. rosea*.

For functional characterization of genes, reverse transcription quantitative PCR (RT‐qPCR) is commonly used to measure the expression levels of candidate genes because of its high sensitivity and throughput, specificity, and accuracy. However, the results are affected by the sample amount, RNA quality and quantity, and efficiencies of cDNA synthesis and PCR amplification (Fleige & Pfaffl, 2006; Udvardi, Czechowski, & Scheible, 2008). Appropriate reference genes are available to eliminate systematic errors and precisely normalize gene expression during RT‐qPCR (Guénin et al., 2009).

However, no reference gene is absolutely consistent in different organisms and their developmental stages; instead, the expression level of a reference gene is stable only under specific conditions. β*‐tubulin* was used to normalize N‐acety1‐β‐D‐glucosaminidase gene in *C. rosea* when confronting with *Fusarium culmorum* (Mamarabadi, Jensen, & Lübeck, 2009), however, it was not available in mycoparasitic process (Sun, Li, & Sun, 2015; Sun, Sun, & Li, 2015). In *Neurospora crassa*, the expression of *act* was not affected by temperature, but it was inhibited under all‐dark conditions (Cusick et al., 2014). Therefore, selection of suitable reference genes under a specific condition is crucial for accurate gene normalization and function certification.

*C. rosea* 67‐1 is an efficient biocontrol fungus active against various plant fungal pathogens (Ma et al., 2011; Zhang, Gao, Ma, & Li, 2004). In previous study, we developed a technique that was able to promote chlamydospore formation in 67‐1 and constructed the transcriptome of chlamydospore formation of the fungus. In this study, nine candidate reference genes—actin (*ACT*), elongation factor 1 (*EF1*), glyceraldehyde‐3‐phosphate dehydrogenase (*GAPDH*), histone (*HIS*), RNA polymerase II CTD phosphatase Fcp1 (*RPP*), succinate‐semialdehyde dehydrogenase (*SSD*), TATA‐binding protein (*TBP*), ubiquitin (*UBQ*), and ubiquitin‐conjugating enzyme (*UCE*) were isolated from *C. rosea* 67‐1 and screened for precise normalization of the differentially expressed genes during the generation of chlamydospores. To our knowledge, this is the first report of reference genes for chlamydospore formation. Our study will provide a basis for further investigation of the genes related to chlamydospore formation and the molecular mechanism underlying chlamydospore formation.

## 2 MATERIALS AND METHODS

### 2.1 Strain

The strain *C. rosea* 67‐1 was originally isolated from a vegetable yard in Hainan Province, China, using the sclerotium baiting method (Zhang et al., 2004). It is preserved in the Biocontrol of Soilborne Diseases Lab of the Institute of Plant Protection, Chinese Academy of Agricultural Sciences.

### 2.2 Production of chlamydospores

*C. rosea* 67‐1 was cultured on potato dextrose agar (PDA) plates at 26°C for 10 days, and the spores were eluted using 10 ml of sterile distilled water and a sterile glass spatula. One milliliter of the spore suspension was inoculated into 50 ml of PD broth in a 250 ml conical flask and incubated at 27°C on a rotary shaker at a speed of 180 r·min^−1^. After 36 hr, the seed liquid was transferred into medium (25 g of glucose, 7 g of soybean cake powder, 0.35 g of urea, 1.0 g of K_2_HPO_4_·3H_2_O, 0.5 g of MgSO_4_, and 50 mg ZnSO_4_·7H_2_O in 1 L water) to produce chlamydospores. The conidiation of *C. rosea* in the medium of corn flour in which no chlamydospores were detected during the whole sampling time was taken as the control. The fungus was incubated at 27°C on a rotary shaker at 180 r·min^−1^, and the thallus were harvested at 36 hr (initial stage of chlamydospore formation) and 72 hr (stage of chlamydospore mass production) after inoculation by using a centrifuge (Sigma 3‐15, Osterode, Germany) at 8000*g* for 15 min and washed three times with sterile distilled water. The residual water was removed using sterile filter paper, and the fresh samples of the fungus were frozen in liquid nitrogen immediately and stored at −80°C. Three completely independent replications were conducted.

### 2.3 RNA extraction and cDNA synthesis

Total RNA of each sample was extracted using the TRIzol reagent (Invitrogen, CA), according to the manufacturer's instructions; RNase‐free DNase I (TaKaRa, Dalian, China) was used to eliminate DNA contamination. RNA integrity was verified using agarose gel electrophoresis, and RNA concentration and quality were detected using a micro‐spectrophotometer (SimpLiNano, Cambridge, UK).

One microgram of the RNA sample was reverse‐transcribed to cDNA in a 20 μl reaction system by using the cDNA FastQuant RT Kit (Tiangen, Beijing, China), and the first‐strand cDNA was maintained at −20°C for real time PCR.

### 2.4 Primer design for the reference genes

On the basis of the transcriptome data for *C. rosea* 67‐1 chlamydospore formation, a total of nine candidates were selected and cloned from the genome of *C. rosea* (Sun, Li et al., 2015; Sun, Sun et al., 2015), namely, *ACT*,* EF1*,* GAPDH*,* HIS*,* RPP*,* SSD*,* TBP*,* UBQ*, and *UCE* (GenBank accession numbers: KP274072, KP274074‐KP274077, KX686112‐KX686115). The primers for the reference genes used for RT‐qPCR were designed using Primer Premier 6 (Table 1), and their specificity was assessed with the following PCR program: 94°C for 3 min, followed by 30 cycles at 94°C for 30 s, 55°C for 30 s, and 72°C for 20 s; final extension at 72°C for 10 min. The amplification efficiency (*E*) of each primer pair was assayed by amplifying twofold serially diluted cDNA templates; the following equation was used: *E* = (10^[−1/slope]^−1) × 100.

**Table 1:** Primers used in this study

Reference gene	Primer sequence (5′‐3′)	Fragment length (bp)	Tm value (°C)	*E*‐value (%)
*ACT* F	CACTCCCGTGACCATTTC	138	55.6	101.1
*ACT* R	AACGAACCAATCCACCCT		54.4	
*EF1* F	TCGATGTCGCTCCTGACT AGCGTGACCGTTTATTTGA	169	53.5	104.6
*EF1* R			54.0	
*GAPDH* F	GGTGCTCGACAGAGTTGC ATCCATTACCCTACATTCCT	109	53.7	109.8
*GAPDH* R			50.4	
*HIS* F	CGCAAGGAGACCTACAGC GACGAAAGAGTTCAGGATGG	102	53.4	99.3
*HIS* R			54.3	
*RPP* F	GCCGTGACGAGAATGGAA CAGGTTTGGGCGGTTCAT	122	57.0	104.9
*RPP* R			58.7	
*SSD* F	TTTCTTCGCCTTTGACACG GCACCTGCTTGCATTCTTG	100	57.0	100.1
*SSD* R			57.3	
*TBP* F	ATCCGTGAACCCAAGACA GCGAGCATATTTGCGTGA	108	53.2	105.5
*TBP* R			56.0	
*UBQ* F	TTACCATCCGCCGACCTG GATCAAACGCACCACCAA	187	59.7	102.7
*UBQ* R			55.0	
*UCE* F	CTCCTATCACGCCACCAA TTCTGTCCATCTGCTCCA	160	54.3	100.8
*UCE* R			51.4	

### 2.5 Reverse transcription quantitative PCR

RT‐qPCR was performed in a 96 well plate with the IQ 5 Multicolor Real‐time PCR Detection System (Bio‐Rad, CA). A 25 μl reaction system was used, and it contained 1 μl of the cDNA template, 1 μl of each forward and reverse primer with the concentration of 10 μmol/L, 9.5 μl of RNase‐free water, and 12.5 μl of 2× SYBR Premix Ex Taq II (TaKaRa, Dalian, China)_._ RT‐qPCR was conducted as follows: denaturation at 95°C for 30 s, followed by 40 cycles at 95°C for 5 s and 55°C for 30 s. The melting curve was generated from 55°C to 95°C every 0.5°C to check the specificity of the primers. Each reaction was performed three times.

### 2.6 Analysis of the stability of gene expression

Three software tools, geNorm (Vandesompele et al., 2002), NormFinder (Andersen, Jensen, & Ørntoft, 2004), and BestKeeper (Pfaffl, Tichopad, Prgomet, & Neuvians, 2004), were used to evaluate the expression stability of the reference genes during chlamydospore generation of *C. rosea* 67‐1. For the geNorm algorithm, the expression stability value *M* was calculated; the gene with the lowest *M* value was regarded to be the most stable, while a higher *M* value indicated lower stability. The pairwise variation value *V* was computed to determine the optimum numbers of reference genes for reliably normalizing gene expressions during 67‐1 chlamydospore formation; the criterion *V*_n/n+1_ ˂ 0.15 was considered as the minimum number of reference genes. Expression stability of the candidate reference genes was estimated based on the analysis of intra‐ and intergroup variation by using NormFinder software; a lower *M* value meant higher stability in gene expression. The BestKeeper software provided standard deviation (*SD*) and coefficient of variance (CV) values calculated from Ct values of the reference genes; lower CV and *SD* values indicated higher stability.

## 3 RESULTS

### 3.1 RNA quality and primer specificity

All RNA samples exhibited two obvious bands of 28S and 18S when agarose gel electrophoresis was performed, and their *A*_260_/*A*_230_ and *A*_260_/*A*_280_ values ranged from 1.9 to 2.2, suggesting high quality for cDNA synthesis.

**Figure 1:**
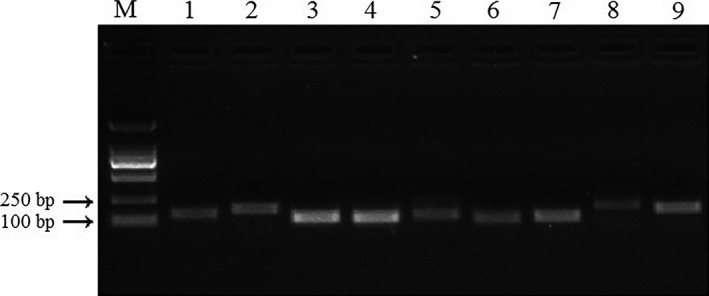
Verification of primer specificity of nine candidate reference genes from *Clonostachys rosea* 67‐1. M: Marker; 1: Actin; 2: Elongation factor 1; 3: Glyceraldehyde‐3‐phosphate dehydrogenase; 4: Histone; 5: RNA polymerase II CTD phosphatase; 6: Succinate‐semialdehyde dehydrogenase; 7: TATA‐binding protein; 8: Ubiquitin; 9: Ubiquitin‐conjugating enzyme

Assessment of the primers showed a single band with an expected size for each candidate in agarose gel electrophoresis (Figure 1) and a single peak in the melting curve, indicating that the primers of the nine reference genes had high specificity and were suitable for the analysis of gene expression. PCR amplification efficiencies of the primers were calculated, and they were in the range of 99%–110% (Table 1).

### 3.2 Expression of the reference genes

**Figure 2:**
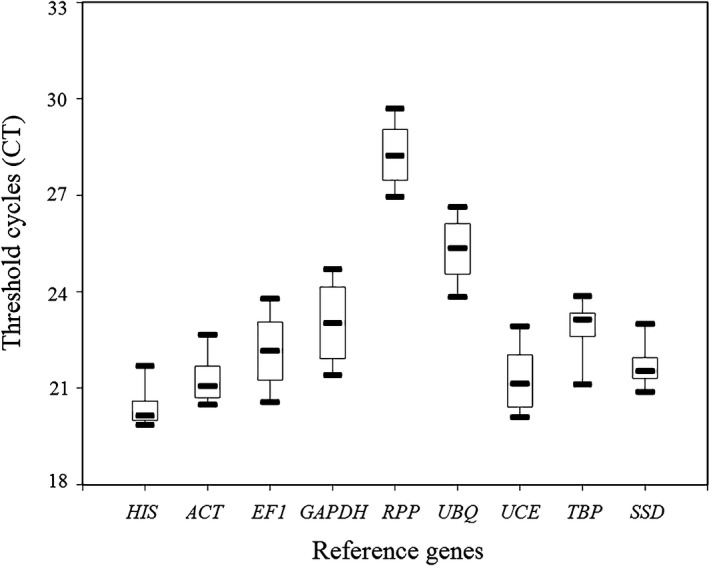
Expression abundance of nine candidate reference genes in *C. rosea* 67‐1 during chlamydospore production. The boxes corresponding to the reference genes indicate the 75th and 25th percentiles, and the horizontal lines in the box‐plots represent the median of three replicates. The bars indicate the maximum and minimum values. HIS, Histone; ACT, Actin; EF1, Elongation factor 1; GAPDH, Glyceraldehyde‐3‐phosphate dehydrogenase; RPP, RNA polymerase II CTD phosphatase; UBQ, Ubiquitin; UCE, Ubiquitin‐conjugating enzyme; TBP, TATA‐binding protein; *SSD*, Succinate‐semialdehyde dehydrogenase

The expression levels of the nine reference genes were reflected by Ct values; *HIS* had the lowest Ct value of 19.9, indicating that the expression levels of the gene were the highest in different sporulation approaches and different stages. *ACT*,* UCE*, and *SSD* exhibited high expressions during *C. rosea* sporulation. *RPP*, with the highest Ct value of 29.7, showed the lowest expression abundance (Figure 2).

### 3.3 Stability of gene expression

#### 3.3.1 geNorm analysis

**Figure 3:**
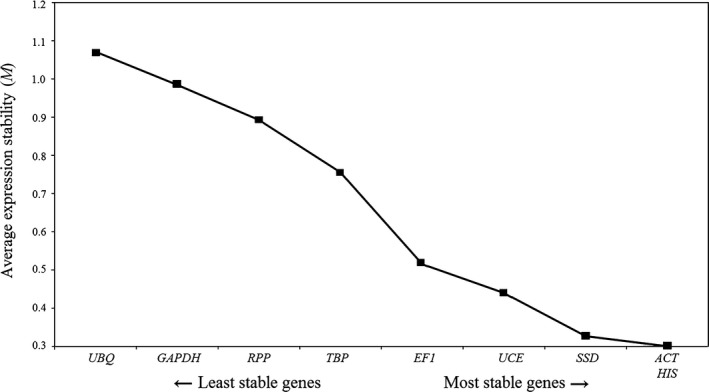
geNorm analysis of expression stability (M) of 9 candidate reference genes in chlamydospore production in *C. rosea* 67‐1. The gene with the lowest *M* value is the most stable, while the gene with the highest *M* value is the least stably expressed. UBQ,Ubiquitin; GAPDH, Glyceraldehyde‐3‐phosphate dehydrogenase; RPP, RNA polymerase II CTD phosphatase; TBP, TATA‐binding protein; EF1, Elongation factor 1; UCE, Ubiquitin‐conjugating enzyme; *SSD*, Succinate‐semialdehyde dehydrogenase; ACT, Actin; HIS, Histone

**Figure 4:**
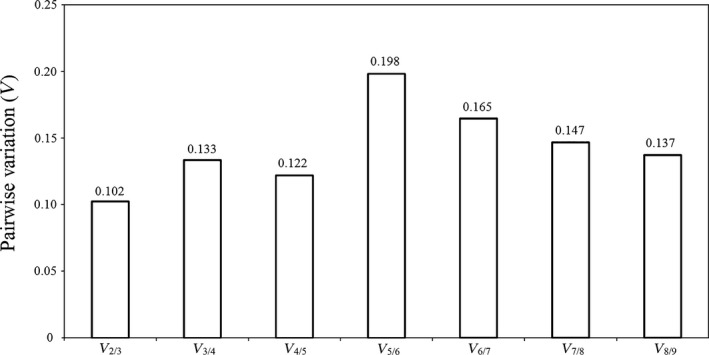
Determination of the optimal number of reference genes during *C. rosea* 67‐1 sporulation by using geNorm analysis. The pairwise variation (*V*_n/n+1_) value was used to estimate the optimal number of reference genes for normalizing gene expression; the value lower than 0.15 indicates that no additional reference gene is required for the normalization of gene expression

The average *M* value of each candidate reference gene was less than 1.5, revealing high expression stability. *ACT* and *HIS* were considered to be the most stable with the lowest *M* value of 0.301, followed by *SSD* with an *M* value of 0.327. *UBQ*, which had a high *M* value of 1.071, was not suitable for gene quantification during *C. rosea* chlamydospore formation (Figure 3). Analysis of pairwise variation yielded *V*_2/3_ = 0.102, indicating that two reference genes should be used to accurately and reliably normalize gene expression during chlamydospore production in *C. rosea* 67‐1 (Figure 4).

#### 3.3.2 NormFinder analysis

Calculation of expression stability values showed that the *M* value of *SSD* was the lowest (0.243), followed by *ACT* (0.406) and *HIS* (0.535); this demonstrated that *SSD*,* ACT*, and *HIS* were stably expressed during chlamydospore production of *C. rosea*. In contrast, *UBQ*, with an *M* value of 0.835, showed a large variation (Table 2).

**Table 2:** Expression stability analysis of nine candidate reference genes by using BestKeeper and NormFinder programs

Reference gene	Bestkeeper^a^		NormFinder
	*SD* [±Ct]	CV [% Ct]	Stability
*ACT*	0.692	3.247	0.406
*EF1*	1.135	5.122	0.601
*GAPDH*	1.288	5.593	0.739
*HIS*	0.616	3.011	0.535
*RPP*	0.984	3.479	0.566
*SSD*	0.629	2.896	0.243
*TBP*	0.849	3.722	0.454
*UBQ*	0.987	3.901	0.835
*UCE*	1.012	4.746	0.575

a *SD* [±Ct] indicates standard deviation of the Ct value; CV [% Ct] represents coefficient of variance expressed as the percentage of the Ct value.

#### 3.3.3 BestKeeper analysis

The expressions of *HIS* and *SSD* were consistent with low *SD* values of 0.616 and 0.629, respectively, while GAPDH, with the highest *SD* value of 1.288, showed relatively low stability during the production of chlamydospores in *C. rosea*.

Based on the results of the three software tools, and according to a comprehensive ranking method described by Wang et al. (2012), the most stable reference gene was *SSD*, followed by *ACT*,* HIS*,* TBP*,* RPP*,* UCE*,* EF1*,* UBQ,* and *GAPDH*. Therefore, *SSD* and *ACT* were finally selected as the optimal reference genes for normalizing gene expression during the generation of chlamydospore in *C. rosea* 67‐1.

## 4 DISCUSSION

RT‐qPCR is an important and widely used method for the quantitative analysis of gene expression in various organisms, and its accuracy and reliable normalization depends on suitable reference genes. Generally, the expression of a reference gene needs to be consistent in all tissues and developmental stages of a species. Therefore, traditional housekeeping genes such as *ACT*,* EF1*,* GAPDH*,* UBQ*, and *UCE* are frequently used as reference genes for RT‐qPCR. However, these genes are not always expressed stably at different periods and treatment conditions. For example, *ACT1* exhibited a significantly lower expression level than the other candidates in *Saccharomyces cerevisiae* under sulfite stress (Nadai, Campanaro, Giacomini, & Corich, 2015), and the variation of *act* expressions in *Trichoderma reesei* was detected when incubated under different conditions (Steiger, Mach, & Mach‐Aigner, 2010). *GAPDH* was also found to be unstable during azole treatment in *Candida glabrata* (Li, Skinner, & Bennett, 2012). Therefore, new reference genes that exhibit stable and high expression levels under specific conditions should be considered. In this study, new candidates such as *RPP* and *SSD* were used to assess their expression stability during chlamydospore formation in *C. rosea* 67‐1.

To explore functional genes in *C. rosea*, several reference genes have been adopted. e.g. *act* was used to normalize ABC transporter genes when suffering to secondary metabolites produced by biocontrol bacteria (Kamou et al., 2016). Zapparata (2014) found that *act* was most stable in different nutrient conditions and glyceraldehyde 3‐phosphate dehydrogenase gene *gpd1* was available when analyzing the interaction with *F. graminearum*. The gene *tub* was also used in the quantification of gene expressions in *C. rosea* when confronting with pathogenic fungi and plant nematodes (Mamarabadi et al., 2009; Zou, Tu, Liu, Tao, & Zhang, 2010). In our previous study, we found that *EF1* was expressed most stably in *C. rosea* 67‐1 during parasitism on *Sclerotinia sclerotiorum* sclerotia, and we obtained a reliable expression profile of mycoparasitism‐related genes by using the reference gene *EF1* (Sun, Li et al., 2015; Sun, Sun et al., 2015). In this study, analyses using geNorm, BestKeeper, and NormFinder showed that the expression of *EF1* was not satisfactory during chlamydospore formation in *C. rosea*. Similar results were obtained during the selection of other reference genes. *MGG_Actin* was proved available to normalize gene expression during the infection of *Magnaporthe oryzae*, but not suitable during fungal vegetative growth (Omar, Bentley, Morieri, & Preston, 2016). Zhou et al. (2012) found that *CrzA* was most stable during the developmental stages of *Beauveria bassiana*, but its stability varied sharply under different stress conditions. The stability of reference genes may vary in response to various environmental conditions, and they may change during the life cycle of an organism; therefore, it is essential to select suitable reference genes for specific conditions.

During transcriptome sequencing and analysis of chlamydospore formation in *C. rosea* 67‐1, we found that some genes, including *SSD*, were consistently expressed at the early stage (36 hr) and stationary phase (72 hr) of sporulation (unpublished). *SSD* is a hydrolase that can catalyze the oxidation of succinic semialdehyde to succinic acid. It is a key enzyme in the catabolism of gamma‐aminobutyric acid (GABA), and it plays a major role in anticonvulsant activity (Kumar, Kumar, & Punekar, 2015; Piplani, Verma, & Kumar, 2016). Deficiency of *SSD* may lead to neurologic disorders (Gahr, Connemann, Schönfeldt‐Lecuona, & Freudenmann, 2013; Lin, Weng, & Lee, 2015; Püttmann et al., 2013). Currently, researchers are focusing on the structure and dynamics of *SSD* (Jang, Park, Chi, & Lee, 2015; Park, Park, & Lee, 2014); however, there is limited information on its expression and variations in organisms. We selected *SSD* and the other candidates to decide whether they could be used as reference genes during fungal sporulation and found that *SSD* was the most stable in chlamydospore production in *C. rosea*, in accordance with a commonly used housekeeping gene *ACT*. To our knowledge, this is the first report of *SSD* as a reference gene for the normalization of gene expression.

In this study, three software tools, geNorm, NormFinder, and BestKeeper, were used to screen the reference genes for quantifying gene expressions during *C. rosea* sporulation. However, differences in the rankings of candidate genes are possible when different statistical algorithms are used (Bansal et al., 2015; Leal et al., 2015). In our study, some differences were observed during the software analyses. geNorm revealed that *ACT* and *HIS* were the most stable, followed by *SSD*. However, NormFinder showed that *SSD* exhibited the highest stability, followed by *ACT* and *TBP*. According to the results of both software tools, *UBQ* and *GAPDH* displayed the largest variation. According to BestKeeper software, *HIS*,* SSD*, and *ACT* were ranked as the top 3 candidate genes, while *GAPDH* and *EF1* expressed with the least stability. However, the trends in general were consistent. To minimize the impacts of the reference genes, a pairwise variation value was calculated based on the expressions of the nine candidate reference genes, and it confirmed that two reference genes were required for reliable and accurate normalization of gene expression. Based on the results, we recommend *ACT* and *SSD* as reference genes for the quantification of gene expression during chlamydospore formation in *C. rosea* 67‐1.

Chlamydospore is of great importance in fungal growth and survival, and it is especially important in the commercialization of biocontrol fungi. To the best of our knowledge, this is the first report on the selection of suitable reference genes for normalizing gene expression during chlamydospore production in a biocontrol fungus. Identification of related genes will facilitate to understand the molecular mechanism underlying chlamydospore formation and promote large‐scale applications of biocontrol agents.

## 5 CONCLUSION

We analyzed the expression stability of nine reference genes during chlamydospore formation in *C. rosea* 67‐1. The results indicated that two reference genes were required for precise normalization of gene expression. *SSD* and *ACT* were the most stable and were selected as reference genes for *C. rosea* sporulation. To the best of our knowledge, this is the first report of screening of sporulation‐related reference genes of biocontrol fungi, and *SSD* was used for the first time as a reference gene. This study will contribute to further investigation of the genes related to chlamydospore production in *C. rosea*.

## CONFLICTS OF INTEREST

None declared.
